# Acute pulmonary embolism treatment in lung transplant recipients: mechanical thrombectomy and catheter directed thrombolysis

**DOI:** 10.1186/s42155-024-00512-z

**Published:** 2025-03-11

**Authors:** Ahmad Arar, Samuel L. Rice, Mhd Wisam Alnablsi, Akhilesh Pillai, Jamaal Benjamin, Rehan Quadri, Daniel Lamus, Anil Pillai

**Affiliations:** 1 Department of Radiology, Interventional Radiology Section, UT Southwestern Medical Center, 5959 Harry Hines Blvd., Professional Office Building I (HP6.600) Mail Code 8834, Dallas, TX 75390-9061 USA; 2 Department of Radiology Plesmanlaan 121, Netherlands Cancer Institute- Antoni Van Leeuwenhoekziekenhuis, 1066 Amsterdam, CX Netherlands; 3 Department of Radiology, Interventional Radiology, University of Texas Health Science Center, 7000 Fannin St, Houston, TX 77030 USA

**Keywords:** Pulmonary embolism, Lung transplantation, Thrombectomy, Catheter-directed thrombolysis, Venous thromboembolism, Deep vein thromboses

## Abstract

**Purpose:**

Acute pulmonary embolism (PE) presents a significant challenge in lung transplant recipients (LTR), even with prophylactic anticoagulation. Due to the heightened risk of complications in this population, the optimal treatment approach for acute PE remains uncertain. This retrospective case series aims to elucidate the outcomes of percutaneous mechanical thrombectomy with the Inari device (MT) and catheter-directed thrombolysis (CDT) in managing acute PE in lung transplant patients.

**Materials and Methods:**

This study examines the treatment outcomes of nine consecutive post-lung transplantation patients with acute PE confirmed with Computed Tomography Angiography (CTA). Treatment interventions included either MT or CDT. Follow-up assessments encompassed a minimum of one year and up to 3 years post-treatment, evaluating various parameters including ICU stay, ventricular pressures, pulmonary function, and laboratory tests.

**Results:**

Both MT and CDT achieved a 100% technical success rate, leading to the successful restoration of pulmonary blood flow and improvements in hemodynamic parameters, with a one-year survival rate of 100%.

**Conclusion:**

Percutaneous treatments, including MT and CDT, demonstrate feasibility and efficacy in managing acute PE among lung transplant patients. These treatments lead to rapid thrombus resolution, post-treatment improvements, and enhanced overall survival.

## Introduction

The European Society of Cardiology (ESC) classifies PE according to early mortality risk into low, intermediate-low, intermediate-high, and high risk, taking into consideration several factors such as hemodynamic instability resulting in hypotension, right ventricular (RV) dysfunction measured with cross-sectional imaging or echocardiography (ECHO), and laboratory markers that signal acute cardiac distress, such as increased cardiac enzyme production [1, 2]. There is limited data regarding the application of these risk stratifications in lung transplantation recipients (LTR). Percutaneous treatments for PE, such as mechanical thrombectomy (MT) and catheter-directed thrombolysis (CDT), have gained popularity for managing higher-risk PE cases, particularly in patients with elevated bleeding risk, who are not candidates for systemic thrombolytics or anticoagulation. Large-bore MT rapidly debulks the thrombus, facilitating the immediate restoration of blood flow to the affected lung, which is particularly important for patients with acute right heart dysfunction [3].

LTRs have an increased risk of developing acute pulmonary embolism (PE), which results in amplified morbidity and mortality. This increased risk is related to several factors: pulmonary compromise due to perfusion abnormalities, pulmonary tissue damage from acute ischemia because the pulmonary artery is the only vascular supply to the lung tissue immediately after transplantation, given the poorly developed bronchial collateral circulation, and vascular collapse resulting from pulmonary hypertension. [4]. Thus, prompt resolution of the PE and restoration of vascular flow to the lungs and pulmonary tissues are advantageous. The appropriate treatment of PE in LTR remains a challenge due to the absence of clear consensus on the optimal treatment approach. The LTR cohort also displays a heightened risk of complications for either medical management with systemic therapy or local interventional procedures especially in the immediate (initial 6 months) post-transplant state.

This case series describes the outcomes of nine patients treated at a single institution for acute PE occurring post lung transplantation (LT), who were managed with either MT or CDT. MT was performed in 6 patients with the INARI device (Inari Medical, Inc., Irvine, CA, USA), a large-bore mechanical thrombectomy catheter for near-instant resolution of clot burden, while the remaining 3 patients underwent CDT using tissue plasminogen activator (tPA) over a 24 h period. The primary goal of both approaches was to promptly restore pulmonary blood flow while minimizing pulmonary tissue damage, the risk of infarction, and long-term complications, as compared to systemic anticoagulation.

## Materials and methods

### Patients

This case series was approved by the Institutional Review Board (IRB) of the University of Texas Southwestern Medical Center. It examines the treatment outcomes of acute PE in nine consecutive LTRs treated between December 2019 and January 2023. Patients were retrospectively identified using electronic medical records. The baseline characteristics and demographics of the patients included are detailed in Table 1. PE diagnosis was confirmed through Computed Tomography Angiography (CTA) in all participants. All participants provided informed consent for medically necessary procedures, adhering to established clinical protocols and standards. Due to the retrospective nature of this investigation, the need for de novo consent was waived by the local ethics committee.

**Table 1:** Baseline patient characteristics and demographics

Patient number	1	2	3	4	5	6	7	8	9
Age (y), gender	62, male	66, male	70, female	65, male	79, male	57, male	55, male	60, male	61, male
Race	W N–H	W N–H	W N–H	W N–H	N–H Latino	W N–H	W N–H	N–H Latino	W N–H
Underlying lung disease	ILD	ILD	ILD	ILD and COPD	ILD and COPD	ILD	ILD	ILD and COPD	ILD
Transplant type	RSL	BLTx	BLTx	LSL	BLTx	BLTx	BLTx	BLTx	BLTx
Surgery or procedure 30 days or less	yes, resection of scalp SCC 5 days before PE	yes, colonoscopy 2 weeks before PE	N/A	N/A	N/A	N/A	N/A	N/A	yes, lung transplant 1 month before PE
Immobilization	yes	yes	no	no	no	no	no	yes	yes
VTE history	yes	no	yes	yes	yes	no	no	yes	no
Cancer	yes (SCC)	no	yes (carcinod tumor)	no	no	no	no	no	no
BMI (kg/m2)	42	31	39	29	27.5	32.91	28.17	33.01	27.07
Pulmonary embolism characteristics									
Size/ location	R main and segmental branches in R upper lobe. Lobar, interlobar, segmental and subsegmental arteries in both R middle and R lower lobes	R and L main and every segmental arteries in both lungs	R main and lobar, segmental, and sub-segmental in all 3 lobes	Proximal L PE present in lobar arteries. Smaller right upper lobe proximal segmental PE	Bil large PE, L and R main arteries	Large straddle PE. Extension in both lobs in L side, with incidental isolated subsegment-al PE in RLL	Bil large central PE	Bil PE, starting at the main pulmonary arteries and extending into the lobar branches and distally	Bil lobar and interlobar branches
Days since transplant	1603	445	392	1260	1612	569	470	1782	36
Allograft affected	yes	yes	yes	yes	yes	yes	yes	yes	yes
Concurrent acute DVT	yes	no	yes	yes	yes	yes	yes	yes	yes
Vasopressor	no	no	no	no	yes	no	no	no	no
HR > 110 bpm	no	no	no	no	yes	no	no	yes	no
RV strain	yes	yes	no	no	yes	no	no	yes	no
PESI score	132	106	140	125	229	97	95	160	81

*Abbreviations:* *W N–H* White Non-Hispanic, *AA* African American, *BLTx* bilateral lung transplant, *BMI* body mass index, *COPD* chronic obstructive pulmonary disease, *DVT* deep vein thrombosis, *ILD* interstitial lung disease, *LSL* left single lung, *PESI* Pulmonary Embolism Severity Index, *RSL* right single lung, *RV* right ventricle, *VTE* venous thromboembolism, *SCC* squamous cell carcinoma, *R* right, *L* left, *Bil* Bilateral, *HR* heart rate, *bpm* beats per minute, *N/A* Not available

### Procedure Technique

Interventional radiologists at a single institution conducted all interventions. All procedures were performed under moderate sedation or monitored anesthesia care (MAC) and involved ultrasound-guided access to the right common femoral vein. For MT, an 8 Fr vascular sheath was placed. A 6 Fr, 110 cm angled pigtail Grollman catheter (Cook Medical, Bloomington, IN, USA) was used to selectively catheterize the pulmonary arteries (PA). Pressure measurements were obtained in all 6 patients. Select angiography of the affected PA was performed in 5 patients. At this point, 3000–5000 units of heparin were administered intravenously, with the dose calculated based on patient weight. For MT, the 8 Fr catheter was removed and over the wire exchanges of vascular sheaths from 10 to 22 Fr were performed, subsequently a 24 Fr DRYSEAL GORE ® (Gore & Associates, Inc., Newark, DE, USA) or 24 Fr Inari sheath was advanced into the inferior vena cava (IVC). Fluoroscopic guidance was used to advance the 20 or 24 Fr Inari catheter thrombectomy device through the heart and into the PA. Thrombectomy was using the Inari, and blood was returned to the patient using the FlowSaver blood return system. Subsequent post-thrombectomy pressure measurements and pulmonary angiography were performed in all patients.

For the CDT procedures, one or two 6 Fr vascular sheaths were placed in the right femoral or jugular vein. Similar technique was used to gain access to the PA. Pressure measurements and selective angiography of PA were performed in all 3 patients. 5 Fr ev3 Cragg McNamara infusion catheter (Medtronic, Minneapolis, MN) was advanced into the affected PA, the sheath and catheters were sutured in place, and a sterile dressing was applied. Patients were then transferred to the medical intensive care unit (MICU), and a total of 1 mg of tPA and 500 units of heparin were administered per hour. Patients underwent labs every 3 h for CBC, PT, PTT, INR, and fibrinogen levels. Standard protocol was used for adjustment of the tPA drip. All CDT patients were returned to the angiographic suit within 24 h for follow-up post-treatment angiography and pressure measurements.

**Fig. 1:**
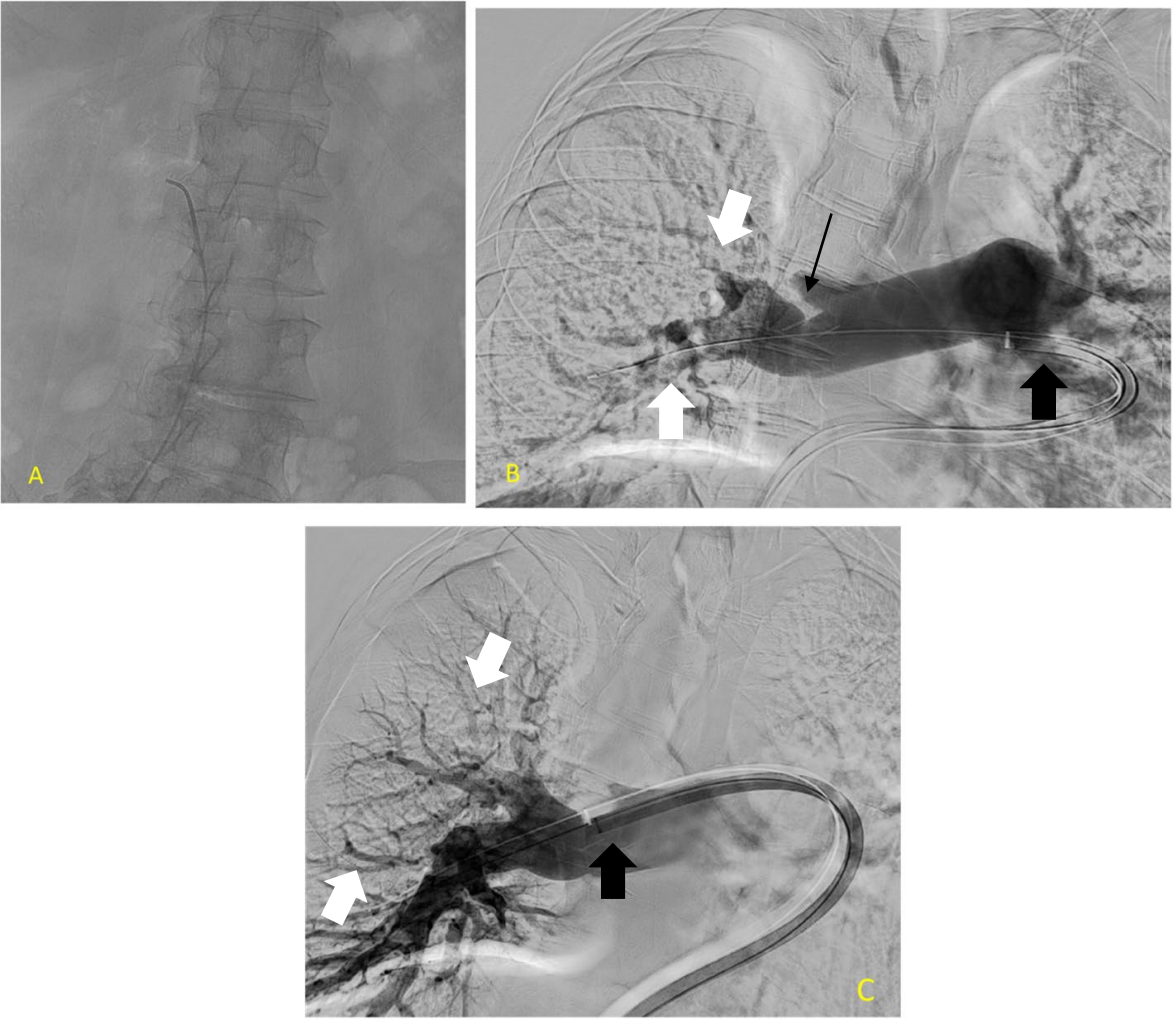
Large bore mechanical thrombectomy with Pulmonary angiogram. **A** Access to the pulmonary artery. **B** Digital subtraction angiogram image demonstrating a pulmonary angiogram performed from the proximal main pulmonary artery (PA) via the Large Bore 24 Fr Inari catheter (Thick black arrow). The thin black arrow identifies the abrupt cut-off of the PA secondary to a thrombus (White arrow). **C** Post large bore MT (Black arrow) angiogram shows restoration of flow through the PA (white arrows)

An IVC filter was inserted during the same MT/CDT procedure in four patients who had Doppler ultrasound verified lower extremity DVT above the popliteal vein. Figure 1 displays fluoroscopic images captured throughout the procedure, illustrating the various steps and highlighting the restoration of pulmonary blood flow following successful treatment. Table 2 describes the procedural details for all patients.

**Table 2:** Procedure Details and Outcomes

Patient number	1	2	3	4	5	6	7	8	9
Date of intervention	10/9/2021	10/14/2020	10/12/2020	12/16/2019	10/1/2022	1/6/2023	1/20/2023	10/31/2022	11/29/2022
Dead/ alive	alive	alive	Dead 11/2023	Dead 02/2023	alive	alive	alive	alive	alive
Procedural duration with IVC filter (minutes)	57	Filter after procedure	No filter	91	Filter after procedure	Filter after procedure	No filter	37	61
Procedural duration without IVC filter (minutes)	42	73	14	79	30	85	135	27	36
Fluoroscopy time (minutes)	16.6	41.4	3.4	12.4	27.8	39.7	45.6	14.9	20.2
Mean pulmonary artery pressure intraprocedural (mmHg)	34	RPA: 26/ LPA: 28	25	30	14	24	18	38	16
Mean pulmonary artery pressure post procedure (mmHg)	13	N/A	23	20	18	19	17	25	10
EBL (ml)	150	10	10	400	50	10	100	10	60
Transfusion (units)	no	no	no	no	yes, 4 RBC and 2 FFP	no	no	no	no
Vital signs									
Preprocedure HR	98	94	85	97	After CPR and ROSC: 120	95	103	122	98
Postprocedure HR at 24 h	72	91	79	81	91	85	85	71	80
Preprocedure BP	117/89	100/89	140/82	123/76	After CPR and ROSC: 83/66	128/93	120/84	87/76	108/77
Postprocedure BP at 24 h	123/83	133/84	133/71	122/70	108/66	134/82	110/76	105/70	122/83
Preprocedural breath frequency	26	19	18	15	20	20	22	20	18
Postprocedure breath frequency	14	20	18	19	18	18	18	17	18
Preprocedural FiO2	21	21	21	21	21	21	N/A	N/A	21
Post procedure FiO2 at 24 h	21	21	21	60	60	21	N/A	N/A	28
Pulmonary function parameters									
FEV1- prior (L)	2.02	3.43	1.65	1.95	2.25	2.96	3.07	1.86	2.3
FEV1-post (1 year)	1.9	3.91	0.89	1.59	2.26	2.91	3.19	1.75	2.39
FEV1-post (1.5 years)	1.91	2.64	1.19	1.59	N/A	N/A	N/A	N/A	N/A
FEV1-post (2 years)	2.09	2.77	1.03	1.55	N/A	N/A	N/A	N/A	N/A
FEV1-post (2.5 years)	1.91	3.08	1.02	N/A	N/A	N/A	N/A	N/A	N/A
FEV1-post (3 years)	N/A	3.04	0.52	N/A	N/A	N/A	N/A	N/A	N/A
FVC-prior (L)	2.52	4.06	2.08	3.15	2.64	4.22	3.56	2.64	3.06
FVC-post (1 year)	2.54	4.59	1.5	2.62	2.63	4.05	3.77	2	3.36
FVC-post (1.5 years)	2.51	3.26	2.13	2.62	N/A	N/A	N/A	N/A	N/A
FVC-post (2 years)	2.65	3.23	1.72	2.49	N/A	N/A	N/A	N/A	N/A
FVC-post (2.5 years)	2.46	3.59	1.71	N/A	N/A	N/A	N/A	N/A	N/A
FVC-post (3 years)	N/A	3.61	0.84	N/A	N/A	N/A	N/A	N/A	N/A
Alive at 1 year	yes	yes	yes	yes	yes	yes	yes	yes	yes
Length of stay (days)	7	7	17	7	21	11	10	8	15
Length of ICU stay (days)	1	3	2	2	5	1	1	4	1
Suppl. O2 pre procedure to maintain spO2 > 90%	6	2	2–3	97% on BiPAP of 12/6 rate and 100%	15	0	0	4	0
Suppl. O2 at discharge to maintain spO2 > 90% at rest	0	0	0	0	0	0	0	2 L on night	0
Suppl. O2 discharge to maintain spO2 > 90% at activity	2	0	0	0–3	0	0	0	2 L on night	0
Suppl. O2 after 6 months	0/ CPAP	0	0 during day, and 2 L at night	0–3 as needed (significant post- transplant events include complication by PGD grade 3)	0	0	Exercise 45 min, 0 L	2 L at night	Exercise 30 min 0 L
Suppl. O2 after 1 year	0/ CPAP	0	2 L PRN and sleep	4	0	N/A	N/A	2 L at night	0
Suppl. O2 after 1.5 years	0/ CPAP	0	2 L PRN and CPAP	5	N/A	N/A	N/A	N/A	N/A
Suppl. O2 after 2 years	0/ CPAP	2–3 L (pseudomonas, covid infection and tracheitis)	2 PRN and CPAP	5	N/A	N/A	N/A	N/A	N/A
Suppl. O2 after 3 years	N/A	0	1–2 L and CPAP	8	N/A	N/A	N/A	N/A	N/A
Saturation pre procedure without O2	82%	86%	88%	67%	Cardiac arrest and CPR: 15 L non-breathing mask to maintain SPO2% > 92%	100% at rest, and 88% on exertion	99% at rest, and 80% on exertion	85%	97%
Saturation post procedure without O2	94%	96%	99%	94%	97%	Improved well with exertion	99% at rest, and improved well on exertion	95%	98%
BNP pre procedure (pg/mL)	210	165	340	1668	1222	45	12	489	144
Troponin Pre (ng/L)	315	228	N/A	N/A	484	7.7	< 5	2063	N/A
Troponin Post 1st (ng/L)	280	212	N/A	N/A	550	N/A	N/A	1566	N/A
Troponin post 2nd (ng/L)	32	48	N/A	N/A	682	N/A	N/A	661	NA

*Abbreviations*: *IVC* Inferior Vena Cava, *RPA* right pulmonary artery, *LPA* left pulmonary artery, *DAP* Dose Area Product, *RBC* red blood cell, *FFP* fresh frozen plasma, *CPR* cardiopulmonary resuscitation, *ROSC* return of spontaneous circulation, *FiO2* fraction of inspired oxygen, *FEV* forced expiratory volume, *FVC* forced vital capacity, *Suppl.* supplemental, *BiPAP* bilevel positive airway pressure, *CPAP* continuous positive airway pressure, *L* liter, *PGD* primary graft dysfunction, *BNP* brain natriuretic peptide, *post 1st* first measurement post-procedure, *post 2nd* second measurement post-procedure, *BP* blood pressure, *EBL* estimated blood loss, *HR* heart rate, *ICU* intensive care unit, *PRN* pro re nata, *N/A* Not available

### Follow-up

All patients underwent follow-up through a review of their medical records until February 2024 (13–39 month follow-up). Clinical parameters recorded included the length of MICU stay, pre- and post-right ventricular pressures, pulmonary artery pressures, left ventricular ejection fraction, RV/LV ratio, supplementary oxygen requirements, pulmonary function test results, and laboratory tests, as detailed in Tables 2 and 3. Follow-up CTA imaging was assessed to evaluate pulmonary infarction and the presence of residual thrombus in the pulmonary arteries.

**Table 3:** Hemodynamic parameters before and after the procedure

Patient number	1	2	3	4	5	6	7	8	9
RV pressure prior (mmHg)	37	48	N/A	26	N/A	N/A	N/A	N/A	34
RV pressure post 1st (mmHg)	N/A	Next day: 26 mmHg	After 36 months: 35 mmHg	32 after 6 months	Next day: 24 mmHg	N/A	28 after 10 months	After 17 months: 25 mmHg	N/A
RV pressure post 2nd (mmHg)	N/A	N/A	N/A	31 after 11 months	After 18 months: 22 mmHg	N/A	N/A	N/A	N/A
LVEF pre	55%	54%	62%	68%	38%	60%	60%	70%	67%
LVEF post 1st	3 days: 55%	5 days: 61%	2 months: 55%	72% after 6 months	Next day: 60%	N/A	10 months: 59%	17 months: 59%	1 week: 53%, 65% after 7 months
LVEF post 2nd	60% after 8 months	56% after 23 months	36 months: 60%	63% after 10 months	18 months: 65%	N/A	N/A	N/A	17 months: 57%
RV/LV pre	4.4/4.6	3.6/3.9	N/A	4.2/4.4	3.5/3.3	3/4	3.1/4.4	N/A	3.6/3.4
RV/LV post 1st	N/A	After 5 days 3.7/4.5	3.6/3.6 after 2 months	3.8/5.5 after 6 months	Next day: 5.1/4.4	N/A	10 months: 3.5/3.9	17 months: 3.3/4.8	2.7/3.9 after 1 week, 7 months: 2.7/4.2
RV/LV post 2nd	N/A	N/A	36 months: 3.6/4	3.3/5.5 after 10 months	18 months: 3/3.7	N/A	N/A	N/A	3.8/3.6 after 17 months

*Abbreviations*: *Post 1st* first measurement post-procedure, *post 2nd* second measurement post-procedure, *RV* right ventricle, *LV* left ventricle, *LVEF* left ventricular ejection fraction, *N/A* Not available

## Results

The age of patients in this study ranged from 55 to 79 years, with a median age of 62 years. The duration between LT and the onset of PE ranged from 36 to 1782 days, with a median time post LT of 569 days. 7 patients underwent bilateral LT, while the other two patients received unilateral LT. All patients exhibited PE involvement in the right and/or left main pulmonary artery or its major branches supplying the allograft. According to the European Society of Cardiology (ESC) criteria, two patients were classified as low-risk (MT: 2), three as intermediate-low risk (MT: 2; CDT: 1), one as intermediate-high risk (MT), and three as massive high-risk PE cases (MT: 1; CDT: 2). The average Pulmonary Embolism Severity Index (PESI) score was 130 (MT: 127; CDT: 135). Notably, patient 5, who had bilateral massive PE, experienced cardiac arrest and required cardiopulmonary resuscitation. Eight patients were hypoxic (MT: 5; CDT: 3), and seven had elevated cardiac biomarker levels (MT: 4; CDT: 3) upon presentation. Detailed demographic attributes and baseline patient characteristics are outlined in Table 1. MT and CDT were technically successful in all patients, without adverse events related to the procedure.

Six patients underwent MT, three of whom had bilateral PE. Procedural durations ranged from 30 to 135 min, and estimated blood loss ranged between 10 and 400 cc. Patient 5 required post-procedural blood transfusions and placement of a chest tube for a hemothorax. Three patients underwent CDT, with total procedure durations ranging from 14 to 73 min and estimated blood loss of 10 cc or less in all cases. After CDT, one patient experienced oozing from the venous access site in the right internal jugular vein with fibrinogen levels below 100 mg/dL. The bleeding was managed with local compression and by adjusting the tPA and heparin doses. In four patients, lower extremity thrombus was identified and IVC filters were placed at the conclusion of the procedure to prevent recurrent PE.

The length of ICU stay ranged from 1–5 days (MT: 1–5; CDT: 2–4), post-procedure, patients exhibited improvement in hemodynamic parameters and respiratory status. The average mean PA pressure was 25 mmHg before treatment and 18 mmHg after treatment, with an average reduction of 6.75 mmHg across all patients; the MT group had an average of 23 mmHg pre- and 16 mmHg post-treatment with an average reduction of 6.5 mmHg, whereas the CDT group had an average of 32 pre- and 24 post-treatment with an average reduction of 7.5 mmHg. The average O^2^ saturation increased from 86% to 96.5% after treatment (MT: 86% pre- to 96% post-treatment; CDT: 86% pre- to 97% post-treatment).

In two patients with diminished left ventricular ejection fractions (LVEF) measured on ECHO (38% and 54%) before treatment, improvements to 60% and 61% were observed 1 day after MT and 5 days after CDT, respectively, on repeat ECHO. In all other cases, measured LVEF was within the normal limit on ECHO pre and post-procedure. Average HR was 101 bpm before treatment and 82 bpm 24 h after (MT: 102 pre- to 82 bpm post-treatment; CDT: 100 pre- to 80 bpm post-treatment). Kidney function, assessed by GFR and creatinine levels, remained stable in all patients with no instances of acute kidney injury detected post-MT/CDT. The one-year survival rate following the procedure (MT or CDT) was 100%. Tables 2 and 3 provide detailed procedural information and post-procedural hemodynamic and respiratory outcomes.

**Fig. 2:**
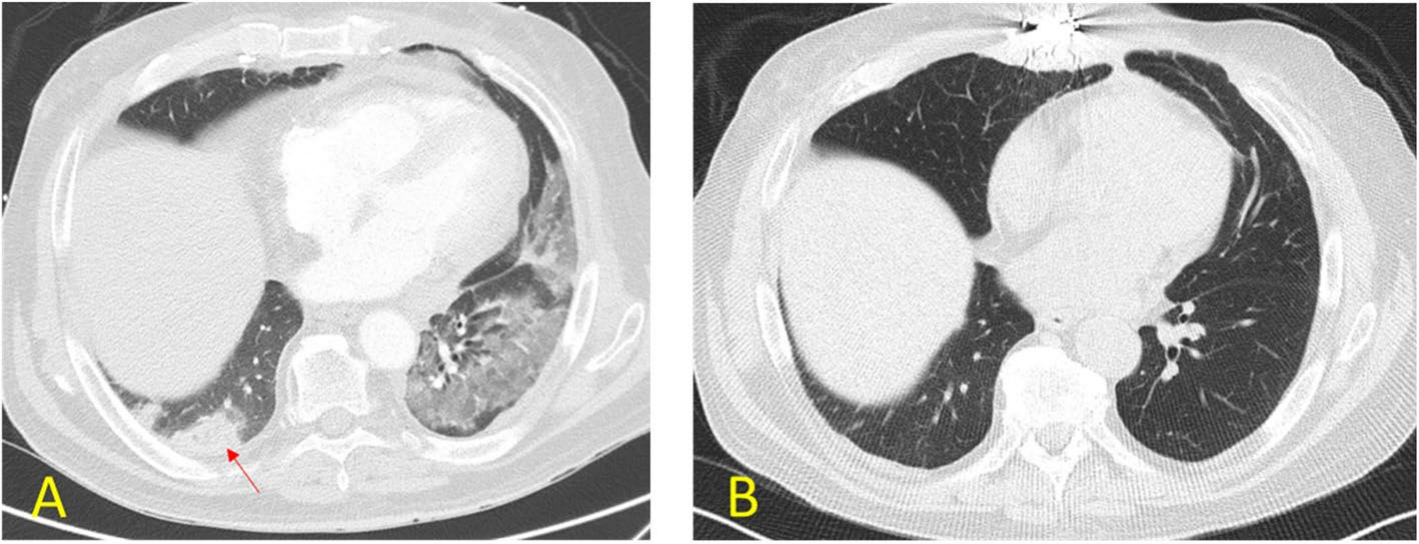
CT scan of the chest before and after thrombectomy in a case of acute PE in a lung transplant patient. 2A: CT scan before thrombectomy showing new ground glass and consolidation involving the lower lobes suggestive of ischemia with development of infarction (Arrow). 2B: CT scan 2 months after thrombectomy showing interval resolution of previously seen infiltrate/consolidation of the lower lobes

Follow-up CTA performed within two months post-procedure was compared to pre-procedure CTA in 7 patients, none of whom showed pulmonary infarction (MT: 4; CDT: 3). All 9 patients had no evidence of infarction on follow-up CTA. One patient who underwent MT initially presented with a right lower lobe pulmonary infarct measuring 2.6 × 5.2 × 5.2 cm (AP x TV x CC). Subsequent imaging two months later in this patient identified near complete resolution of the pulmonary infarction; instead, a small consolidative opacity in that region was observed (refer to Fig. 2). The average FEV1 one year post-LT was 2.31 L, compared to 2.39 L pre-LT, with continued improvement in FEV1 observed in some patients after 1 year. FVC was similar pre-LT (3.1 L) and 1 year post-LT (3.0 L), and increased in some patients after 1 year. Basic laboratory tests were monitored both before and after treatment, with Table 4 displaying the most significant test results.

**Table 4:** Lab results before and after the procedure

Patient number	1	2	3	4	5	6	7	8	9
GFR pre (mL/min)	36	> 60	47	49	45	33	43	50	> 60
GFR post procedure (mL/min)	54	> 60	47	58	39	34	42	> 60	> 60
GFR at discharge (mL/min)	43	> 60	41	58	48	30	42	> 60	> 60
Creatinine pre (mg/dL)	1.9	1.32	1.29	1.81	1.5	2.27	2.01	1.58	1.04
Creatinine post procedure (mg/dL)	1.42	1.13	1.2	1.7	1.76	2.21	1.85	1.12	0.84
Creatinine at discharge (mg/dL)	1.72	1.27	1.37	1.41	1.48	2.48	1.86	0.87	1.14
WBC pre (per microL)	7430	8310	5130	16,300	10,590	5980	5490	12,970	7310
WBC post procedure (per microL)	6560	6340	6560	11,620	12,030	4760	4330	9070	5350
WBC at discharge (per microL)	5830	10,070	8660	7510	8290	6190	8840	8980	5280
Hgb pre (g/dL)	12.4	15.2	9.4	10.9	12.4	11.5	12.3	14.9	11.9
Hgb post procedure (g/dL)	10.9	13.3	10	9.2	7.7	8.9	9.1	12.8	11.3
Hgb at discharge (g/dL)	10.2	14.5	10	8.6	12.1	8.7	10	12.9	12.2

*Abbreviations*: *GFR* Glomerular filtration rate, *WBC* White blood cells, *Hgb* Hemoglobin

## Discussion

Venous thromboembolism (VTE) remains a significant concern among LTRs despite the implementation of prophylactic anticoagulation measures. Incidence rates of VTE in this population range from 9 to 43%, with the majority occurring within the initial 30 days post-surgery [5]. This carries a notable risk for acute PE and is correlated with unfavorable prognosis and reduced survival rates post-LT [6, 7]. The reported incidence of PE post-LT varies from 5 to 15% [7]. A recent study by Zheng et al. implemented a universal screening approach after LT utilizing venous duplex scans across all extremities. In the first year after transplantation, 24.7% of recipients developed VTE. Of these cases, 24% had PE and 76% were isolated DVTs. This cohort's overall one-year survival rate post-VTE was 86.1% [5]. Thus, the use of PE risk scale employed in patients without LT, has limitations when applied to LTR with PE.

Two other studies have examined the prevalence of PTE in patients who died within 1 year after LT through autopsy evaluations. The first study reported PE in 13% of the cases, while the second study found a prevalence of 29%, with 67% of PE-related deaths occurring within the first month. This suggests that both the prevalence and mortality rate of PE after LT may be underestimated [8, 9]. The lack of collateral bronchial supply in the allograft increases the risk of pulmonary infarction, especially if the treatment is delayed, potentially predisposing patients to an increased risk of respiratory failure. This can lead to hypoxia and right ventricular dysfunction with hemodynamic compromise that can justify the elevated rates of morbidity and mortality [4, 10]. Additionally, some LTRs may have poorer baseline lung function, further enhancing their susceptibility to adverse outcomes when PE occurs [11]. Such considerations underscore the significant impact of PE on LTRs and highlight how percutaneous catheter-directed treatments can have an active role in rapidly extracting or dissolving thrombi, thereby reducing the risk of ischemia.

Management usually encompasses heparin-based anticoagulation followed by oral vitamin K antagonist. Insertion of an IVC should be considered in the appropriate context [12]. Utilizing anticoagulation and thrombolysis after LT as prophylactic management presents a challenge in treating PE in LT patients due to the increased risk of hemorrhage. Percutaneous catheter-directed interventions are increasingly employed for managing intermediate- and high-risk PE cases, particularly those with elevated bleeding risks. The utilization of large-bore MT with catheters ranging from 16 to 24 Fr has shown promising results in PE patients displaying imaging evidence of right ventricular (RV) dysfunction. These procedures expedite thrombus removal, promptly reinstating blood flow to the affected lung [3]. On the other hand, Efthymios et al. conducted a meta-analysis encompassing 20 studies involving 1168 patients, revealing that CDT exhibits high success rates in both high- and intermediate-risk PE cases. However, in high-risk PE cases, it is anticipated that there will be heightened risks of complications, including elevated rates of bleeding and mortality [13]. In this series, we describe the outcomes after Large bore MT and CDT in the treatment of 9 lung transplant patients who developed acute PE. Both procedures were successful without adverse effects. Immediately after MT the mean PA pressure decreased from 23 to 16 mmHg, with an average decrease of 6.5 mmHg. Mean oxygen saturation also increased from 86 to 96% post procedure. On the second day of evaluation after CDT, mean PA pressure decreased from 32 to 24 mmHg, with an average decrease of 7.5 mmHg, and mean oxygen saturation increased from 86 to 97%. Mean hemoglobin concentration decreased from 11.9 to 9.5 g/dL post MT and from 13.1 to 12 g/dL post CDT. The ICU stay for MT ranged from 1 to 5 days, while for CDT it ranged from 2 to 4 days. The average duration from admission to discharge was 11.8 days for MT and 10.6 days for CDT. Our experience showed that both treatments were effective in preventing pulmonary infarction in LTRs with PE due to the rapid restoration of pulmonary blood flow. This, along with immediate improvements in pulmonary artery pressure, oxygen saturation, and hemodynamic parameters, was beneficial to this patient population and contributed to a 100% survival rate at one year.

Risk factors for developing PE post-LT can be categorized into pretransplant, intraprocedural, and post-transplant factors. These encompass older age, elevated body mass index (BMI), prior VTE, prolonged mechanical ventilation, pre-and/or post-transplant extracorporeal membrane oxygenation (ECMO), intraoperative cardiopulmonary bypass (CPB), extended intensive care unit (ICU) stay following surgery, early-onset infections, prolonged duration of central venous catheter (CVC) indwelling, and interruption of VTE prophylaxis within the initial five days post-transplantation [5, 14]. One randomized controlled trial comparing tacrolimus-sirolimus-prednisone (TAC/SIR/prednisone) with tacrolimus-azathioprine-prednisone (TAC/AZA/prednisone) immunosuppressive regimens post-transplantation demonstrated a higher incidence of VTE among recipients receiving sirolimus compared to azathioprine [15]. Many of these risk factors were present in our cases: the average age was 64 years; 5 patients had a prior history of VTE, 5 had a BMI > 30, 8 had concurrent acute DVT, 4 had recent surgery or immobilization, and 2 had active cancer. The duration between LT and the development of PE varied widely in our study with an average of 569 days.

While data is limited, these techniques could hold particular significance in treating acute PE in patients with LT. A recent study described the use of MT in treating 8 LT patients with intermediate and high-risk proximal PE, reporting a post-procedure survival rate of 87% at one month [11]. Limitations of this study include the small sample size, the widely variable duration between LT and PE diagnosis, the absence of a control group, and the lack of quality-of-life metrics, which may impact the generalizability of the results. Although MT and CDT are feasible and promising for LTRs, further studies are needed to validate these results.

## Conclusion

In conclusion, this study demonstrates the feasibility and efficacy of percutaneous treatments (MT and CDT) for managing acute PE in LTRs. Both interventions achieved rapid restoration of pulmonary blood flow and enhanced hemodynamic parameters, resulting in a 100% one-year survival rate. Follow-up evaluations showed no pulmonary infarction and improvements in chest CTAs. These findings highlight the importance of continuous monitoring to optimize outcomes in LTRs with acute PE.

## Data Availability

The datasets used and/or analyzed during the current study are available from the corresponding author upon reasonable request.
